# *IRGM/Irgm1* facilitates macrophage apoptosis through ROS generation and MAPK signal transduction: *Irgm1*^+/-^ mice display increases atherosclerotic plaque stability

**DOI:** 10.7150/thno.62797

**Published:** 2021-09-09

**Authors:** Shaohong Fang, Song Sun, Hengxuan Cai, Xiaoyi Zou, Shanjie Wang, Xinran Hao, Xin Wan, Jiangtian Tian, Zhaoying Li, Zhongze He, Wei Huang, Chenchen Liang, Zhenming Zhang, Liming Yang, Jinwei Tian, Bo Yu, Bo Sun

**Affiliations:** 1 Department of Cardiology, Second Affiliated Hospital of Harbin Medical University, Harbin 150086, China.; 2 The Key Laboratory of Myocardial Ischemia, Chinese Ministry of Education, Harbin 150086, China.; 3 Department of Neurobiology, Harbin Medical University; Neurobiology Key Laboratory, Education Department of Heilongjiang Province, Harbin 150086, China.; 4 Department of Pathophysiology, Harbin Medical University-Daqing, Daqing 163319, China.

**Keywords:** *IRGM/Irgm1*, macrophage, apoptosis, atherosclerosis, plaque stability

## Abstract

**Rationale**: Atherosclerosis plaque rupture (PR) is the pathological basis and chief culprit of most acute cardiovascular events and death. Given the complex and important role of macrophage apoptosis and autophagy in affecting plaque stability, an important unanswered question include is whether, and how, immunity-related GTPase family M protein (IRGM) and its mouse orthologue IRGM1 affect macrophage survival and atherosclerotic plaque stability.

**Methods:** To investigate whether serum IRGM of ST-segment elevation myocardial infarction (STEMI) patients is related to plaque morphology, we divided 85 STEMI patients into those with and without plaque rupture (PR and non-PR, respectively) based on OCT image analysis, and quantified the patients' serum IRGM levels. Next, we engineered *Irgm1* deficient mice (*Irgm1*^+/-^) and chimera mice with *Irgm1* deficiency in the bone marrow on an *ApoE*^-/-^ background, which were then fed a high-fat diet for 16 weeks. Pathological staining was used to detect necrotic plaque cores, ratios of neutral lipids and cholesterol crystal, as well as collagen fiber contents in these mice to characterize plaque stability. In addition, immunofluorescence, immunohistochemical staining and western blot were used to detect the apoptosis of macrophages in the plaques. *In vitro*, THP-1 and RAW264.7 cells were stimulated with ox-LDL to mimic the *in vivo* environment, and IRGM/IRGM1 expression were modified by specific siRNA (knockdown) or *IRGM* plasmid (knocked-in). The effect of *IRGM/Irgm1* on autophagy and apoptosis of macrophages induced by ox-LDL was then evaluated. In addition, we introduced inhibitors of the JNK/p38/ERK signaling pathway to verify the specific mechanism by which *Irgm1* regulates RAW264.7 cell apoptosis.

**Results:** The serum IRGM levels of PR patients is significantly higher than that of non-PR patients and healthy volunteers, which may be an effective predictor of PR. On a high-fat diet, *Irgm1*-deficient mice exhibit reduced necrotic plaque cores, as well as neutral lipid and cholesterol crystal ratios, with increased collagen fiber content. Additionally, macrophage apoptosis is inhibited in the plaques of *Irgm1*-deficient mice. *In vitro*, *IRGM/Irgm1* deficiency rapidly inhibits ox-LDL-induced macrophage autophagy while inhibiting ox-LDL-induced macrophage apoptosis in late stages. Additionally, *IRGM/Irgm1* deficiency suppresses reactive oxygen species (ROS) production in macrophages, while removal of ROS effectively inhibits macrophage apoptosis induced by IRGM overexpression. We further show that *Irgm1* can affect macrophage apoptosis by regulating JNK/p38/ERK phosphorylation in the MAPK signaling pathway.

**Conclusions:** Serum IRGM may be related to the process of PR in STEMI patients, and *IRGM/Irgm1* deficiency increases plaque stability. In addition, *IRGM/Irgm1* deficiency suppresses macrophage apoptosis by inhibiting ROS generation and MAPK signaling transduction. Cumulatively, these results suggest that targeting *IRGM* may represent a new treatment strategy for the prevention and treatment of acute cardiovascular deaths caused by PR.

## Introduction

Worldwide, acute coronary syndrome (ACS) is the leading cause of death and disability, and atherosclerosis is its pathological basis 1, 2. Postmortem studies revealed that plaque rupture (PR) causes up to 75% of ACS episodes 3. Hence, timely and accurate identification of vulnerable plaque are important new directions in the treatment of coronary atherosclerosis, which will significantly help patients at very high risk (i.e., >15% acute coronary events per year) 4.

Recently, optical coherence tomography (OCT) in coronary arteries has enabled the identification of plaque morphology 5. In our previously study, vulnerable plaques are characterized by a large necrotic core with a thin overlying fibrous cap via OCT analysis 6. In addition to imaging features, mounting evidence has indicated that macrophage death (including apoptosis and autophagy) is a crucial determinant of lesion stage and plaque stability 7-9. In early atherosclerotic lesions, macrophage foam cells undergo autophagy, a regulated 'recycling process' that redirects metabolic pathways 10. Besides, lipid-loaded macrophages undergo apoptosis and are effectively efferocytosed 11. However, in advanced atherosclerotic lesions, autophagy is dysfunctional and apoptotic cells are also not efficiently efferocytosed, leading to inflammation and necrotic core formation 12. However, unlike the feasibility of necrotic core (NC) size assessment by imaging, there is no available clinical method to accurately assess the degree of inflammation and cell death in atherosclerotic plaques 12.

IRGM, a human immunity-related GTPase 13, 14, was recently highlighted as a regulator of inflammatory homeostasis in the intestine, which is seemingly related to its emerging role in autophagy 15-17. However, these studies yielded conflicting conclusions. In cells that interfere with *IRGM* and the murine homologue *Irgm1*, the autophagy process induced by IFN-γ and starvation is impaired 18. Contrary to these earlier reports, introduction of a tandem fluorescent reporter gene into *Irgm1*-deficient mice revealed its dispensable role in the early stages of autophagosome biogenesis 19. We previously found that *IRGM/Irgm1* is highly expressed in the macrophages of patients with atherosclerosis and is essential for actin-dependent CD36 mediated ox-LDL uptake by macrophages 20. In addition, we found that *IRGM/Irgm1* inhibits macrophage polarization to the pro-inflammatory M1 phenotype 21. However, it remains unknown whether *IRGM/Irgm1* affects macrophage survival and plaque stability during atherosclerosis.

Reactive oxygen species (ROS)-induced lipid peroxidation plays a critical role in cell apoptosis and autophagy 22. For instance, autophagy has a protective role by eliminating ROS 23. However, ROS can also induce excessive autophagic cell death 24. In addition, there are two primary alternative pathways (the extrinsic and intrinsic pathways) that lead to apoptosis, both of which can be stimulated by lipid peroxidation 25, 26. Caspases are key molecules involved in transduction of the two apoptosis signals, and finally converging at the executioner caspase3 27. Meanwhile, mitogen-activated protein kinases (MAPKs) represent the main cellular signal transducers in response to oxidative stress 28, 29. Moreover, the product of lipid peroxidation forms adducts with signal-regulated kinase (ERK), p38, and Jun N-terminal kinase (JNK) to activate MAPKs, ultimately activating caspase signal initiation of the apoptotic processes 30, 31. Meanwhile, studies in patient-derived tissues 32 and animal models 33 have demonstrated that MAPK-deficiency in macrophages leads to increased macrophage apoptosis and atherosclerosis progression 34. However, different studies produced seemingly contradictory results 35, 36. Therefore, it is necessary to investigate the role of *IRGM/Irgm1* on ROS and MAPK signaling pathways, which will further elucidate the mechanism by which it regulates macrophage survival.

Herein, we classified patients with ST-segment elevation myocardial infarction (STEMI) according to the presence of PR and found that serum IRGM levels are closely related to PR in STEMI patients. Subsequently, we clarify that *Irgm1* deficiency can effectively increase plaque stability and macrophage apoptosis. Importantly, we investigated the molecular mechanism underlying *Irgm1* regulating macrophage apoptosis. *Irgm1* deficiency can inhibit ROS production and MAPK signaling pathway activation, ultimately inhibiting macrophage apoptosis. These findings strongly suggest that *IRGM/Irgm1* might serve as a marker for fragile plaque during atherosclerosis as well as a therapeutic target.

## Results

### Serum IRGM is correlated with atherosclerotic PR events in STEMI patients

**Figure 1:**
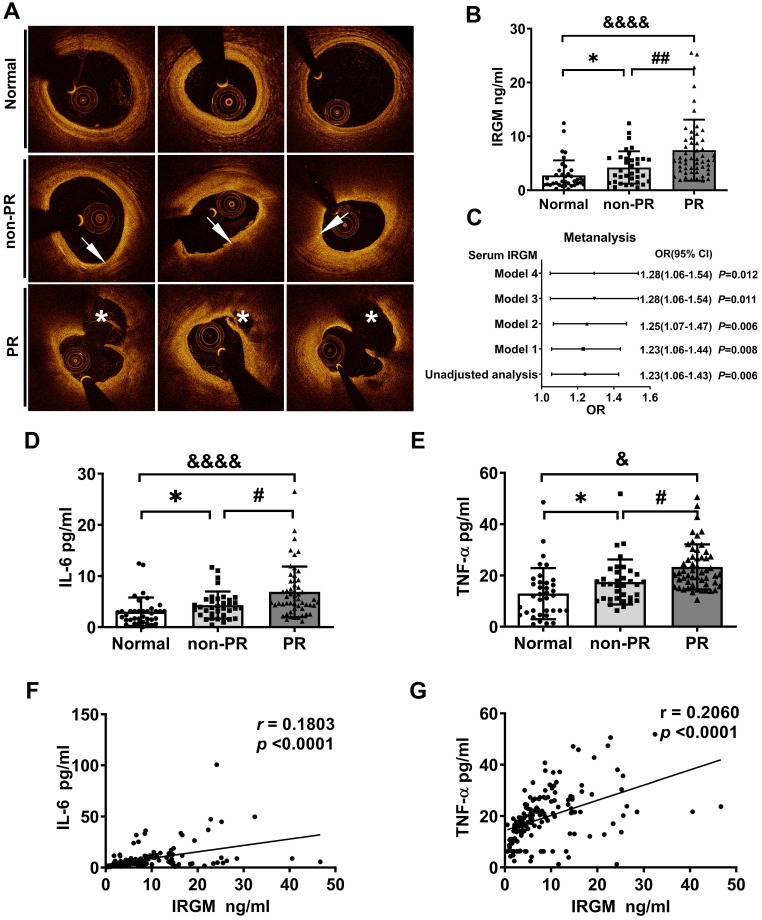
** IRGM is associated with atherosclerotic plaque rupture (PR) in patients with STEMI.** (A) Representative OCT images in normal coronary vessels, non-PR plaques, and PR plaques; arrows show TCFA, with FCT < 65 μm, and lipid arc > 90°; asterisks represent ruptured cavities at the culprit site. (B) Serum IRGM levels among healthy volunteers, non-PR patients, and PR patients. *p < 0.05; ##*p* < 0.01; &*p <* 0.05. (C) Adjusted ORs and 95% CIs for PR associated with serum IRGM in patients with STEMI by four models: Model 1, adjusted for age; Model 2, in which Model 1 was further adjusted for hypertension, diabetes mellitus, and current smoking; Model 3, in which Model 2 was additionally adjusted for lipid factors (LDL-C and TC) and Model 4, in which Model 3 was further adjusted for peak TnI and hs-CRP. (D) Serum IL-6 levels among healthy volunteers, non-PR patients, and PR patients as detected by ELISA. *p < 0.05; ##*p* < 0.01; &*p <* 0.05. (E) Serum TNF-α levels among healthy volunteers, non-PR patients, and PR patients as detected by ELISA. *p < 0.05; #*p* < 0.05; &*p* < 0.05. (F) Quantitative data of the correlation between serum IRGM and IL-6 in PR patients. (G) Quantitative data of the correlation between serum IRGM and TNF-α in PR patients.

First, we investigated whether serum IRGM is related to plaque morphology in STEMI patients. Baseline clinical characteristics between the PR and non-PR groups were compared in Table S1. The serum LDL-C and total cholesterol (TC) levels in the PR group were significantly increased, while other indicators were unaffected. According to OCT image analysis (Table S2), 85 STEMI patients were divided into patients with and without plaque rupture (PR (n = 52; 61.2%) and non-PR (n = 33; 38.8%), respectively). An additional 43 healthy volunteers (Normal) were also included (Figure S1). Three sets of representative OCT images were shown in Figure 1A. Subsequently, the serum IRGM levels were quantified. Results showed that the PR group had the highest serum IRGM level, which was significantly higher than that in the non-PR group and healthy volunteers (Figure 1B). After fully adjusting the covariates, IRGM was still an effective PR predictor (OR 1.28 95% CI 1.06~1.54, p = 0.012; Figure 1C). Besides, considering that TNF-α and IL-6 play pivot roles in PR progression 37, we quantified the serum IRGM, IL-6 and TNF-α expression (Figure 1D, E). Results showed that the PR group had the highest serum IL-6 and TNF-α levels, while serum IRGM content was positively correlated with IL-6 and TNF-α abundance (Figure 1F, G). These results suggest that serum IRGM may be related to the process of PR in STEMI patients.

### Irgm1 deficiency increased mouse atherosclerotic plaque stability

**Figure 2:**
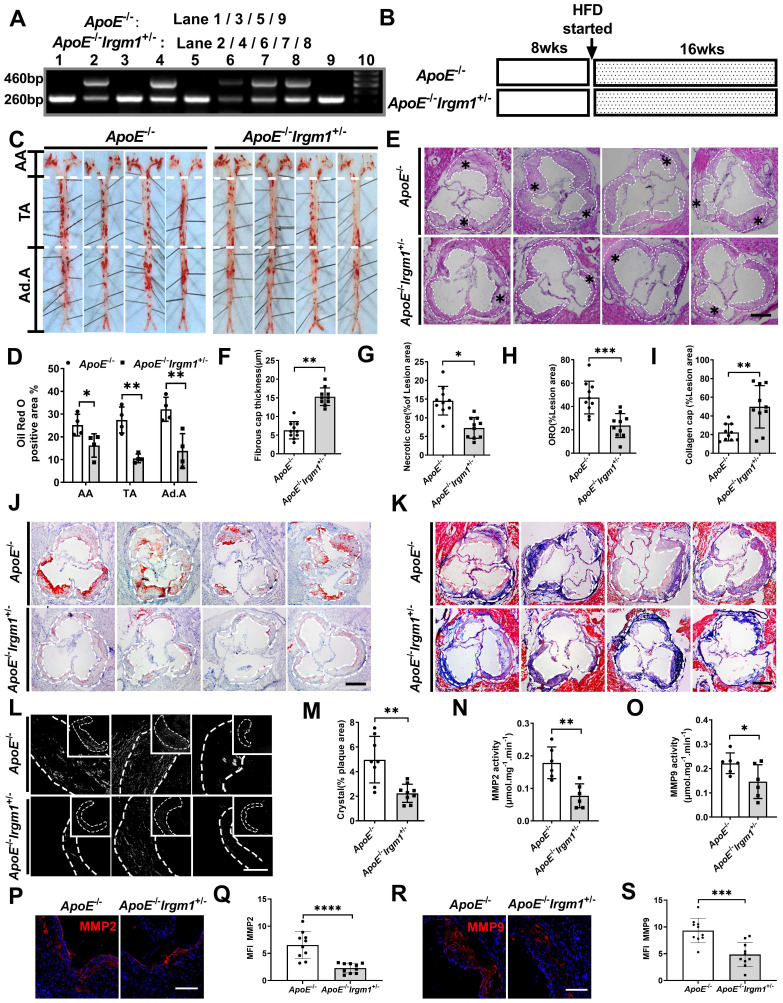
*** Irgm1* deficiency increases mouse atherosclerotic plaque stability. (A)** Genotype analyses for IRGM; Lanes 1, 3, 5 and 9 represent *ApoE*^-/-^ mice, Lanes 2, 4, 6, 7 and 8 represent *ApoE*^-/-^*Irgm1*^+/-^ mice. **(B)** Study design for establishment of advancing atherosclerotic plaques in mice. After a high-fat diet for 16 weeks, mice were euthanised and frozen or paraffin slices of tissues prepared for staining. HFD, high-fat diet; wks, weeks. **(C, D)** Representative images and quantitative analyses of ORO staining in three parts of the aorta: aortic arch (AA) (n = 4), thoracic aorta (TA) (n = 4), and abdominal aorta (Ad.A) (n = 4). **(E)** Representative images for H&E staining to assess the FCT, necrotic lipid core, and plaque areas in the aortic sinus (n = 10). Necrotic lipid cores are denoted by *; black dashed lines indicate the contour of the plaques; scale bars: 200 µm. **(F)** Quantitative analyses area and thickness of FCT (n = 10). **(G)** Percentage of necrotic core areas in the plaques (n = 10). **(J)** Neutral lipid was detected in the aortic sinus by ORO staining (n = 10); scale bars: 200 µm. **(K)** Representative images for the detection of collagen in the aortic sinus as revealed by Masson staining (n = 10); scale bars: 200 µm. **(H, I)** Quantitative analyses of the percentage of neutral lipid and collagen content in the plaques, respectively. **(L)** Representative images of cholesterol crystals observed by confocal microscopy (n = 8~9); scale bars: 50 and 100 µm. **(M)** Quantitative analyses of the proportion of cholesterol crystals in the plaque. **(N-O)** After a high-fat diet for 16 weeks, MMP2 (N) and MMP9 (O) activities of *ApoE*^-/-^ and *ApoE*^-/-^*Irgm1*^+/-^ mice aortic tissue were quantified. **(P, R)** Representative images of MMP2(P) and MMP9(R) observed by confocal microscopy after 16 weeks of a high-fat diet in *ApoE*^-/-^ and *ApoE*^-/-^*Irgm1*^+/-^ mice aortic sinus; nuclei were stained with DAPI (blue). (n = 8~9); scale bars: 100 µm. **(Q, S)** Quantitative data represent the MMP2(Q) and MMP9(S) mean fluorescence intensity in plaque of *ApoE*^-/-^ and *ApoE*^-/-^*Irgm1*^+/-^ mice. *p < 0.05, **p < 0.01, ***p < 0.001, ****p < 0.01. Results are presented as the mean ± SD. Statistical analysis: unpaired Student's *t*-test.

To study the role of *Irgm1 in vivo*, we first distinguished *ApoE*^-/-^*Irgm1*^+/-^ and *ApoE*^-/-^ mice through genotype analysis (Figure 2A). We confirmed that compared with *ApoE*^-/-^ mice, *ApoE*^-/-^*Irgm1*^+/-^ mice had significantly reduced *Irgm1* mRNA levels in 11 major organs or murine tissues, including the aorta, heart and bone marrow cells (Figure S2A). Then, *ApoE*^-/-^*Irgm1*^+/-^ and *ApoE*^-/-^ mice were fed a high-fat diet for 16 weeks, after which aortic lesions were analysed (Figure 2B). Compared with *ApoE*^-/-^ mice, the proportion of ORO positive areas in the lumen regions of the *ApoE*^-/-^*Irgm1*^+/-^ mouse aortic arch, thoracic aorta, and abdominal aorta were significantly reduced (Figure 2C, D). Meanwhile, *ApoE*^-/-^*Irgm1*^+/-^ mice had thicker fibrous caps and a lower NC ratio (Figure 2E-G). Which further verified our conclusion, compared with *ApoE*^-/-^ mice, neutral lipid content and cholesterol crystals in *ApoE*^-/-^*Irgm1*^+/-^ mice were significantly reduced (Figure 2H-K), while the ratio of collagen significantly were significantly increased (Figure 2L, M). Furthermore, the expression of MMP2 and MMP9 in the aortic sinus in *ApoE*^-/-^*Irgm1*^+/-^ mice were significantly reduced (Figure 2P-S). Consistent with this, the MMP2 and MMP9 activities in the aortic tissue of *ApoE*^-/-^*Irgm1*^+/-^ mice was also significantly reduced (Figure 2N, O). In addition, no significant difference was observed in serum lipid levels between the two groups both before and after a high-fat diet for 16 weeks (Figure S2B-E). These results indicate that Irgm1 deficiency increased mouse atherosclerotic plaque stability.

### IRGM/Irgm1 regulates macrophage autophagy in response to ox-LDL *in vitro* is an early-stage event

**Figure 3:**
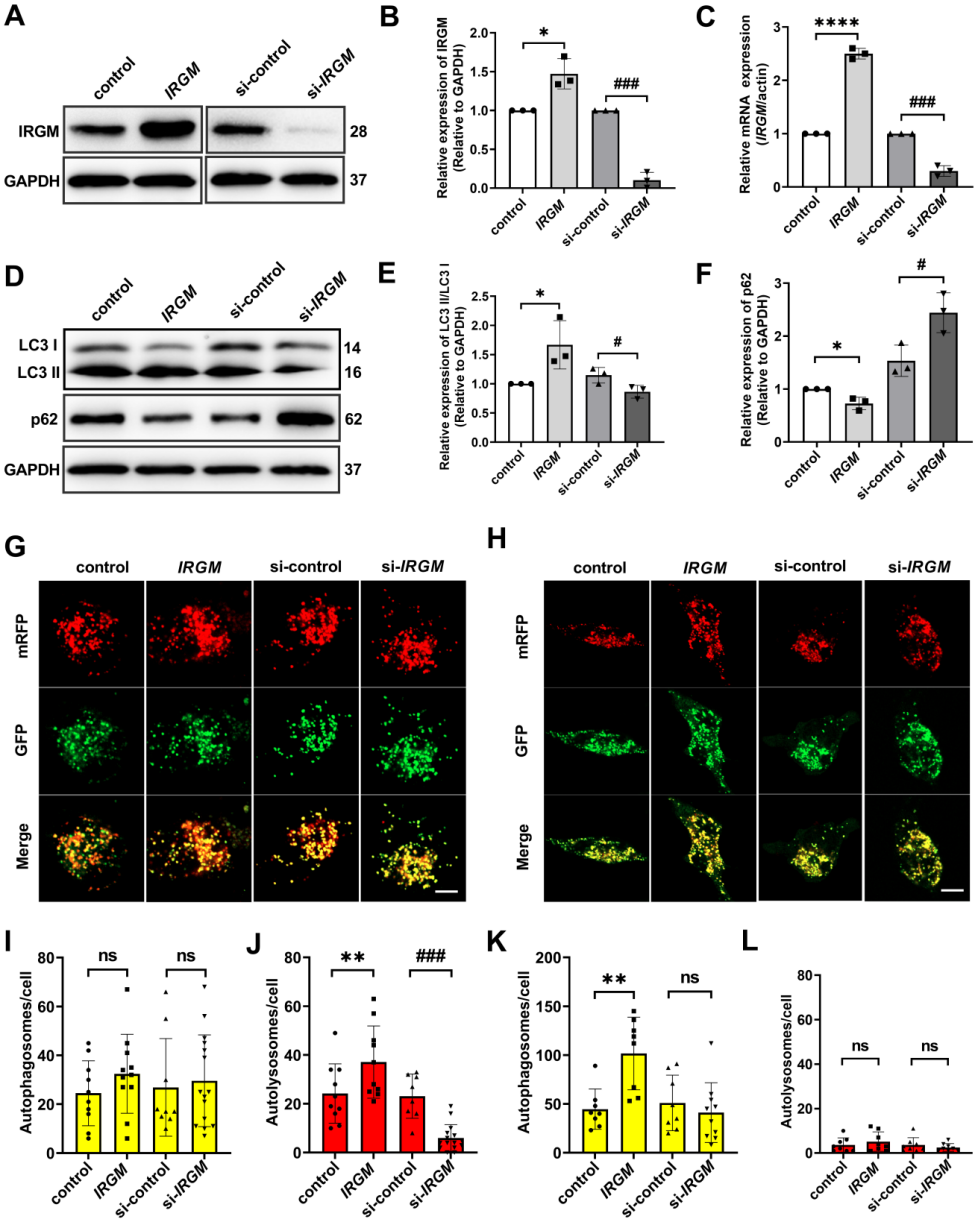
*** IRGM/Irgm1* regulates macrophage autophagy in response to ox-LDL *in vitro* is an early-stage event. (A-F)** THP-1 cells were transfected with either control or IRGM plasmids (Lanes 1, 2) or transduced with either control or IRGM siRNA (Lanes 3, 4) and then stimulated with ox-LDL (50 µg/mL) for 3 h. The WB (A) and Qpcr (C) were used to detect the transfection efficiency and quantitative analysis results are shown (n = 3) (B). Quantitative data represent the fold change after normalised to GAPDH. **(D-F)** The expression of autophagy-related proteins LC3 and P62 were detected by western blot. **(G)** After stimulating THP-1 cell with ox-LDL (50 μg/mL) for 3 h, the autophagy double-labelled adenovirus fluorescent probe was used to detect the expression of autophagosomes (yellow) and autophagolysosomes (red). Green fluorescence was quenched in an acidic environment. Scale bar: 10 µm. **(I, J)** Quantitative analysis of the yellow and red puncta in (G). **(H)** After stimulating THP-1 cell with ox-LDL (50 μg/mL) for 48 h, the autophagy double-labelled adenovirus fluorescent probe was used to detect the expression of autophagosomes (yellow) and autophagolysosomes (red). Green fluorescence is quenched in an acidic environment. Scale bar: 10 µm. **(K, L)** Quantitative analysis of yellow and red puncta in (H). *p < 0.05, **p < 0.01, ****p < 0.0001, #*p* < 0.05, ###*p* < 0.001. Results are presented as mean ± SD. Statistical analysis: unpaired Student's *t*-test.

Macrophage can eliminates ox-LDL through autophagy in some circumstance 38. Considering the important role of macrophage survival in PR, we further hypothesized that *IRGM/Irgm1* might regulate macrophage autophagy. First, IRGM/IRGM1 expression were modified by specific siRNA (knockdown) or *IRGM* plasmid (knock-in) (Figure 3A-C, Figure S3A-C). Western blot results showed that after ox-LDL (50 μg/mL) treatment for 3 h, compared with control, overexpression of *IRGM* significantly increased LC3II/I and decreased p62. In contrast, knocking down *IRGM/Irgm1* significantly decreased LC3 II/I and increased p62 (Figure 3D-F, Figure S3E, F). Meanwhile, knocking down *Irgm1* significantly reduced mRNA levels of autophagy-related genes *Atg5, Atg7, Beclin1*, and *LC3* (Figure S3D). Furthermore, we used mRFP-GFP tandem fluorescently labelled LC3II to evaluate autophagosome-lysosome fusion. RFP-positive/GFP-negative puncta indicate fusion. Compared with control, after ox-LDL treatment for 3 h, overexpression of *IRGM* significantly increased RFP-positive/GFP-negative spots, while knocking down *IRGM/Irgm1* significantly reduced RFP-positive/GFP-negative spots (Figure 3G, I, J, Figure S3G, I). Results indicate that *IRGM* promotes macrophages to complete the autophagic flux in response to ox-LDL in the early-stage, while *IRGM/Irgm1* deficiency suppresses macrophages to complete the autophagic flux. However, after ox-LDL treatment for 48 h, with or without *IRGM/Irgm1* modification, only RFP-positive/GFP-positive spots were observed (Figure 3H, K, L, Figure S3H, J). Results indicate that,* in vitro,* in response to ox-LDL, *IRGM/Irgm1* does not regulate macrophages to complete the autophagic flux in late-stage.

### IRGM/Irgm1 deficiency suppresses macrophage apoptosis in response to ox-LDL and in atherosclerotic lesions

**Figure 4:**
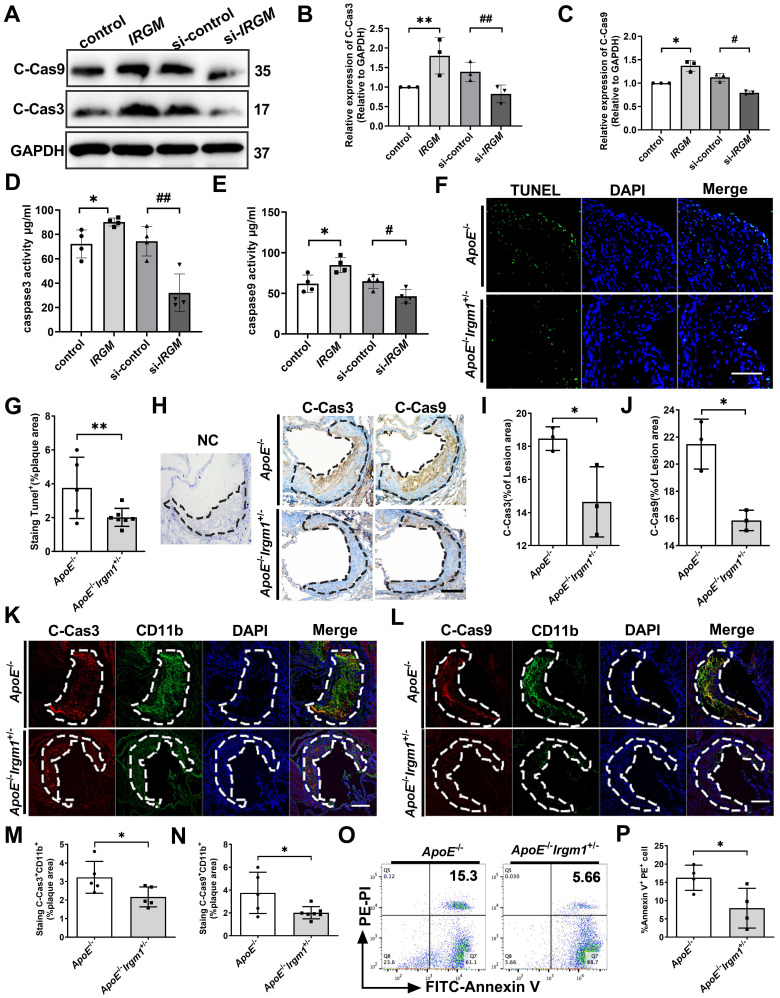
** IRGM/*Irgm1* deficiency suppresses macrophage apoptosis in response to ox-LDL in atherosclerotic lesions. (A-E)** THP-1 cells were transfected with either negative control or IRGM plasmids (Lanes 1, 2), or transduced with either control or IRGM siRNA (Lanes 3, 4) and then stimulated with ox-LDL (50 µg/mL) for 48 h. **(A-C)** Western blotting was used to detect the expression of cleaved-caspase3/9 (A), and quantitative analysis results are shown (B, C). GAPDH was used as a loading control. Quantitative data represent the fold change after normalised to GAPDH. **(D-E)** Caspase3 (D) and caspase9 (E) activities were quantified. **(F)** TUNEL^+^ cell areas in aortic sinus plaques from *ApoE*^-/-^ (n = 3) and *ApoE*^-/-^*Irgm1*^+/-^ mice (n = 3). Areas circled by dashed lines represent the contour of plaques; scale bars: 200 µm. **(G)** Quantification of the percentage of TUNEL^+^ areas within the plaques. Representative images (H) and quantitative analyses **(I, J)** of cleaved-caspases 3/9 in the plaques (n = 3); NC, negative control; scale bars: 100 µm. **(K-P)** *ApoE*^-/-^ and *ApoE*^-/-^*Irgm1*^+/-^ mice were given a high-fat diet for 16 weeks. **(K)** Representative images for co-location of cleaved-caspase3 (red) and CD11b (green) by immunofluorescence staining (n = 3 in each group). **(L)** Co-location of cleaved-caspase9 (red) and CD11b (green) by immunofluorescence staining (n = 3 in each group); nuclei stained by DAPI (blue); scale bars: 200 µm. **(M, N)** Quantitative data of the co-location percentage of CD11b^+^ with cleaved-caspase3 (M) and cleaved-caspase9 (N). **(O, P)** Peritoneal macrophages were recruited, and then FITC-Annexin V and PE-PI were co-stained and quantified by flow cytometry (n = 4 in each group). *p < 0.05, **p < 0.01, #*p* < 0.05, ##*p* < 0.01. Results are presented as the mean ± SD. Statistical analysis: unpaired Student's *t*-test.

In addition to disrupting the autophagic process, ox-LDL reportedly induces apoptosis through cell death receptors and mitochondrial pathways, leading to NC formation 39-41. Therefore, we examined the impact of *IRGM/Irgm1* on macrophage sensitivity to apoptosis* in vitro*. The results showed that after ox-LDL treatment for 48 h, compared with the control, *IRGM*-overexpressing THP-1 cells had significantly increased levels of cleaved-caspase3/9, the activated form of this apoptotic effector protein (Figure 4A-C). In contrast, compared with the si-control, knocking down *IRGM* significantly decreased cleaved-caspase3/9 (Figure 4A-C). Similar results of caspase3/9 activities were also detected in THP-1 cells (Figure 4D, E). In addition, after ox-LDL treatment for 48 h, knocking down *Irgm1* in RAW264.7 cells also significantly decreased the expression of cleaved-caspase3/9 and caspase3/9 activity (Figure S3K-N). These results indicate that in response to ox-LDL, *IRGM/Irgm1* deficiency decreased macrophage apoptosis in the late-stage *in vitro*.

Next, *in vivo*, *ApoE*^-/-^*Irgm1*^+/-^ mice had a significantly decreased proportion of TUNEL-positive (TUNEL^+^) cells in atherosclerotic lesions compared to *ApoE*^-/-^mice (Figure 4F, G). Meanwhile, *ApoE*^-/-^*Irgm1*^+/-^ mice had a significantly decreased proportion of cleaved-caspase3-positive (C-Cas3^+^) and cleaved-caspase9-positive (C-Cas9^+^) cells in atherosclerotic lesions (Figure 4H-J). Importantly, in *ApoE*^-/-^*Irgm1*^+/-^ mice atherosclerotic lesions, co-localization of CD11b and cleaved-caspase3/9 were significantly decreased (Figure 4K-N). Compared with *ApoE*^-/-^mice, the apoptosis of peritoneal macrophages in *ApoE*^-/-^*Irgm1*^+/-^ mice was also significantly reduced (Figure 4O, P). Collectively, these results indicate that *IRGM/Irgm1* deficiency suppresses macrophage apoptosis in response to ox-LDL *in vivo and vitro*.

### IRGM/Irgm1 deficiency suppresses ROS-induced macrophage apoptosis

**Figure 5:**
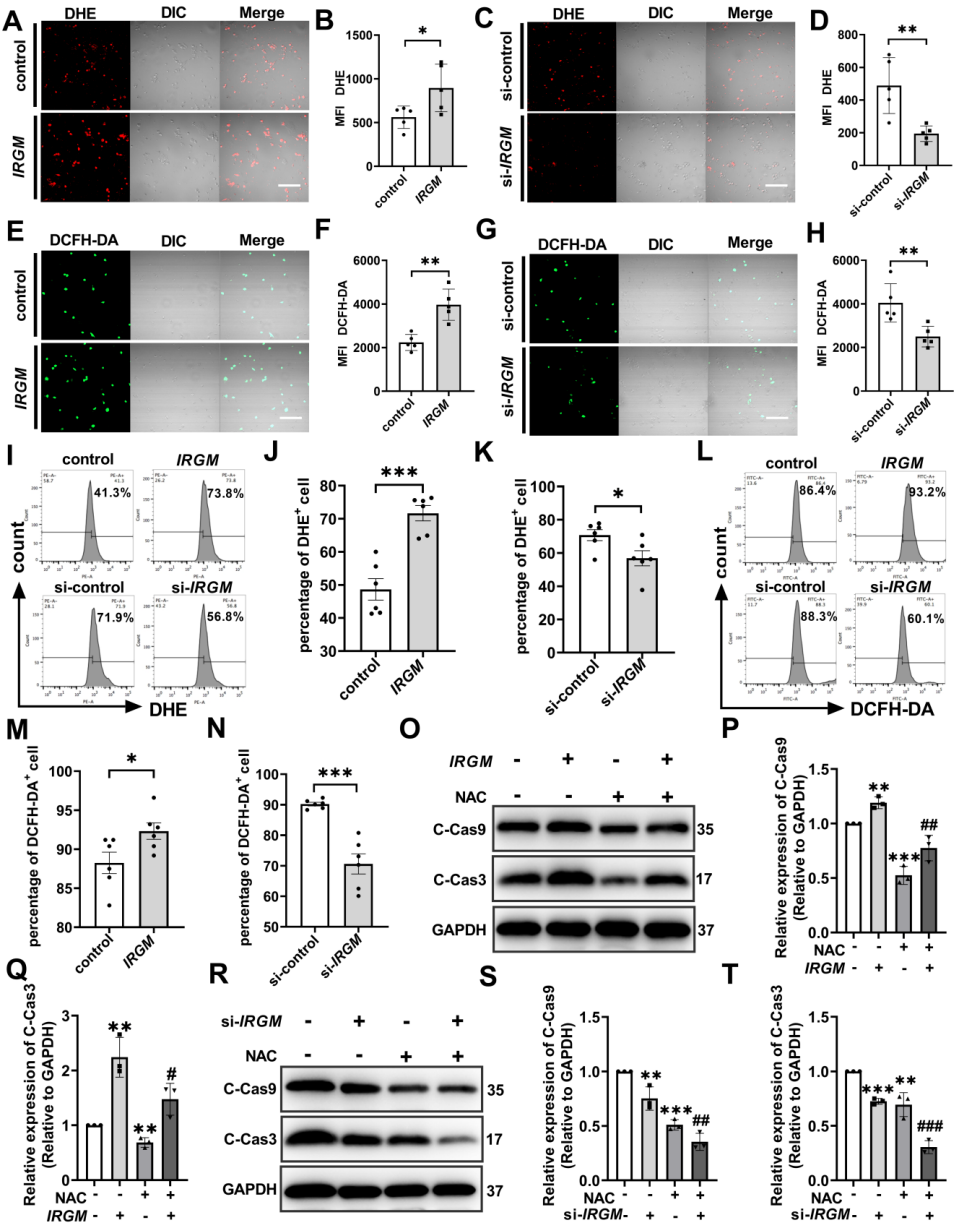
***IRGM/Irgm1* deficiency suppresses ROS-induced macrophage apoptosis.** THP-1 cells were transfected with either negative control or IRGM plasmids, or transduced with control or IRGM siRNA and then stimulated with ox-LDL (50 µg/mL) for 48 h. **(A, C, E, G)** Representative images of the reactive oxygen species labelled with DHE (5 µM) or DCFH-DA (5 mg/mL) fluorescent probe were observed by confocal microscopy (n = 3 per group); scale bars: 50 µm. **(B, D, F, H)** Quantitative data in the graph represent relative mean fluorescence intensity (MFI) (n = 3 per group). *p < 0.05, **p < 0.01. **(I, L).** Flow cytometry was used to detect ROS labelled with DHE (5 µM) or DCFH-DA (5 mg/mL) fluorescent probes (n = 6 per group). **(J, K, M, N)** Quantitative data represent the percentage of DHE^+^ and DCFH-DA^+^ macrophages. *p < 0.05, ***p < 0.001. **(O, R)** Western blotting was used to detect the expression of cleaved-caspase3/9. GAPDH was used as a loading control. **(P, Q, S, T)** Quantitative data represent the fold change after normalisation to GAPDH. vs control, *p < 0.05, **p < 0.01; vs IRGM, #*p* < 0.05, ##*p* < 0.01, ###*p* < 0.001. Results are presented as the mean ± SD. Statistical analysis: unpaired Student's *t*-test.

ROS is the key executor of oxidative stress, which causes apoptosis during atherosclerosis 42, 43. First, to determine the effect of *IRGM/Irgm1* on ROS production, two fluorescent probes with different specificities i.e. dihydroethidium (DHE) and 5-(and-6)-chloromethyl-2',7'-dichlorodihydrofluorescein diacetate (DCFH-DA) were employed. Compared with the control group, the fluorescence intensity of DHE and DCFH-DA in THP-1 cells increased significantly when IRGM was overexpressed (Figure 5A, B, E, F). In contrast, compared with si-control, the fluorescence intensity of DHE and DCFH-DA were significantly reduced when *IRGM* was knocked down (Figure 5C, D, G, H), with similar results observed in RAW264.7 cells (Figure S5A, B). In addition, flow cytometry analysis revealed a significant increase in DHE and DCFH-DA positive THP-1 cells following overexpression of *IRGM,* while the opposite effect was observed following *IRGM* knockdown (Figure 5I-N); consistent results were obtained in RAW264.7 cells (Figure S5C, D). These results suggest that *IRGM* promotes ROS production, and *IRGM/Irgm1* deficiency decreases ROS production in macrophages.

Further, we assessed the effects of *IRGM/Irgm1* on ROS-induced macrophage apoptosis. Compared with the control and si-control group, the active oxygen scavenger N-acetylcysteine (NAC) significantly decreased the abundance of cleaved-caspase3/9 in THP-1 cells (Figure 5O-T). Notably, IRGM overexpression in THP-1 cells caused a significant increase in cleaved-caspase3/9, whereas NAC reversed this effect (Figure 5O-Q). Meanwhile, knocking down *IRGM* and NAC synergistically decreased cleaved-caspase3/9 in THP-1 cells significantly (Figure 5R-T). Consistent results were observed in *Irgm1*-knockdown RAW264.7 cells (Figure S5E-H). Finally, TUNEL staining positive cells in the si*-Irgm1* group were also significantly reduced, and NAC further reduced positive cells proportion (Figure S5I, J). Collectively, these results suggest that *IRGM/Irgm1* deficiency suppresses ROS production and ROS-induced macrophage apoptosis.

### Irgm1 deficiency suppresses apoptosis in macrophages induced by MAPK signaling pathway activation

**Figure 6:**
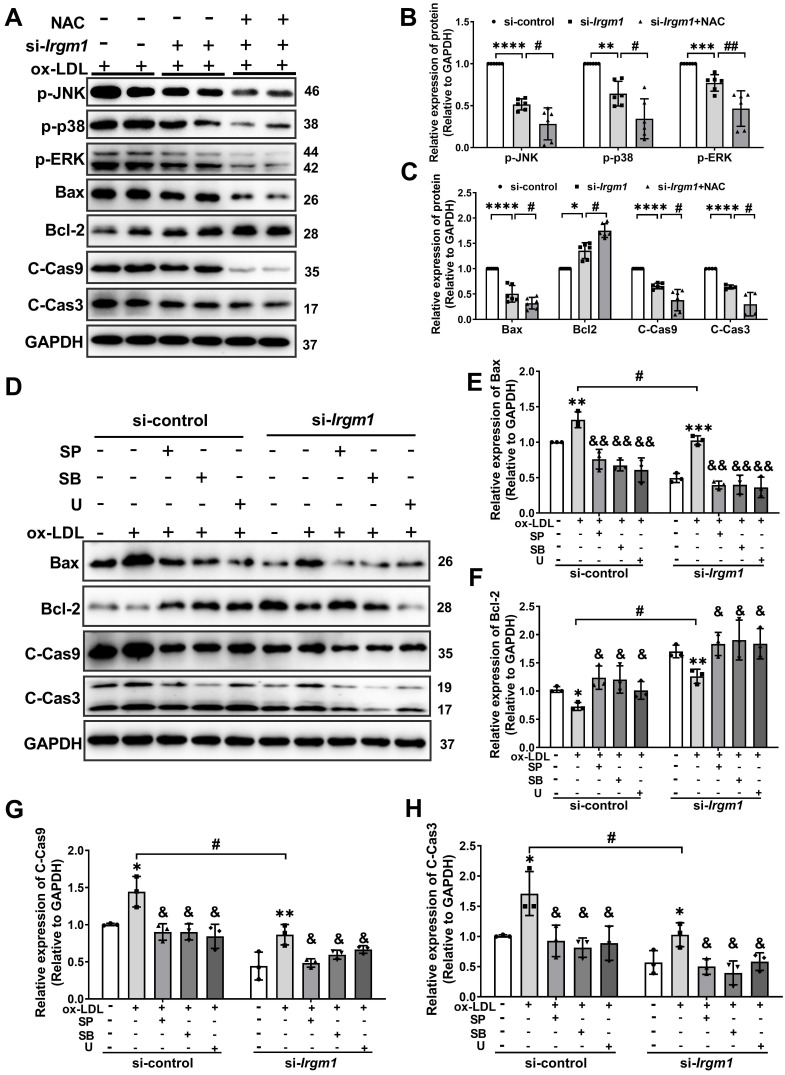
***Irgm1* deficiency suppresses apoptosis in macrophages induced by MAPK signaling pathway activation. (A-C)** The Raw 264.7 cells were transfected with si-*Irgm1* and si-control, in the presence or absence of NAC, and then stimulated with ox-LDL (50 µg/mL) for 48 h. Representative image (A) and quantitative data (B-C) of p-JNK, p-38, p-ERK, and apoptosis-related Bax, Bcl-2, and cleaved-caspase3/9 protein levels. GAPDH was used as a loading control. Quantitative data representing the fold change after normalised to GAPDH. si-control vs. si-*Irgm1*: *p < 0.05, **p < 0.01, ***p < 0.001, ****p < 0.0001; si-*Irgm1* vs. si-*Irgm1*+NAC, #*p <* 0.05, ##*p <* 0.01. **(D)** Expression of Bax, Bcl-2, and cleaved-caspase3/9 were detected by Western blotting in si-control (Lanes 1-5) and si-*Irgm1* (Lanes 6-10) groups, after treatment with SP600125 (10 μM), SB203580 (10 µM) or U0126 (10 µM) for 3 h. GAPDH was used as a loading control. **(E-H)** Quantitative data representing the fold change after normalisation to GAPDH.; Lane 2 vs. Lane 1 and Lane 6 vs. Lane 7: *p < 0.05, **p < 0.01, ***p < 0.001; Lane 3, 4, 5 vs. Lane 2; Lane 8, 9, 10 vs. Lane 7, &*p <* 0.05, &&*p <* 0.01; Lane 7 vs. Lane 2: #* p <* 0.05. Results are presented as the mean ± SD. Statistical analysis: unpaired Student's* t-*test.

MAPK signaling has been implicated in apoptosis in response to ROS 44. To define the molecular mechanisms responsible for the *Irgm1* effect on macrophage apoptosis, we knocked out *Irgm1* in RAW264.7 cells with siRNA, which were then treated with ox-LDL for 48 h. The phosphorylation of JNK, p38 and ERK were then used to characterize MAPK signaling pathway activity. Results showed that compared with si-control, the levels of p-JNK, p-p38, and p-ERK in the si-*Irgm1* group were significantly decreased (Figure 6A lanes 1-4; Figure 6B, C). Simultaneously, the key apoptosis proteins Bax, cleaved-caspase9, and cleaved-caspase3 in the si-*Irgm1* group were also significantly decreased, while Bcl-2 was significantly increased (Figure 6A, lanes 1-4; Figure 6B, C). Importantly, NAC showed synergistically effects with si-*Irgm1 in* MAPK signal and apoptosis related proteins (Figure 6A, lane 5, 6; Figure 6B, C). These results indicate that the suppression of the JNK/p38/ERK signal transduction, together with ROS production, serve as the primary features in *Irgm1*-deficent macrophage apoptosis.

To further verify the role of the *Irgm1 in* macrophage apoptosis induced by MAPK signaling activation, we pretreated RAW264.7 cells with JNK, p38, and ERK inhibitors SP600125 (SP), SB203580 (SB), and U0126 (U), respectively, and then added ox-LDL and siRNA (Figure S6A-F). Compared with treatment with ox-LDL alone, regardless of the inhibitor or si-*Irgm1* treatment decreased JNK, p38, and ERK phosphorylation significantly (Figure S6A-F). Moreover, si-*Irgm1* acted synergistically with the inhibitors, further significantly reduced phosphorylation of JNK, p38, and ERK (Figure S6A-F). Next, we examined the expression of key apoptosis proteins (Figure 6D-H). The results showed that, ox-LDL treatment resulted in a significant increase in the expression of Bax, cleaved-caspase9 and cleaved-caspase3, while Bcl-2 decreased (Figure 6D Lanes 1, 2, 6, 7; Figure 6E-H). Whereas, compared with ox-LDL stimulation, treatment with inhibitors significantly reversed above effects (Figure 6D lanes 2 and 3-5; lanes 7 and 8-10; Figure 6E-H). More importantly, si-*Irgm1* acted synergistically with the inhibitors, further reduced the expression of above key apoptosis proteins (Figure 6D lanes 2-5; lanes 7-10; Figure 6E-H). Collectively these results suggest that *Irgm1* deficiency suppresses apoptosis in macrophages induced by MAPK signaling pathway activation, and ROS acts as an upstream event.

### Irgm1 deficiency in haematopoietic cells increases mouse atherosclerotic plaque stability and suppresses macrophage apoptosis

**Figure 7:**
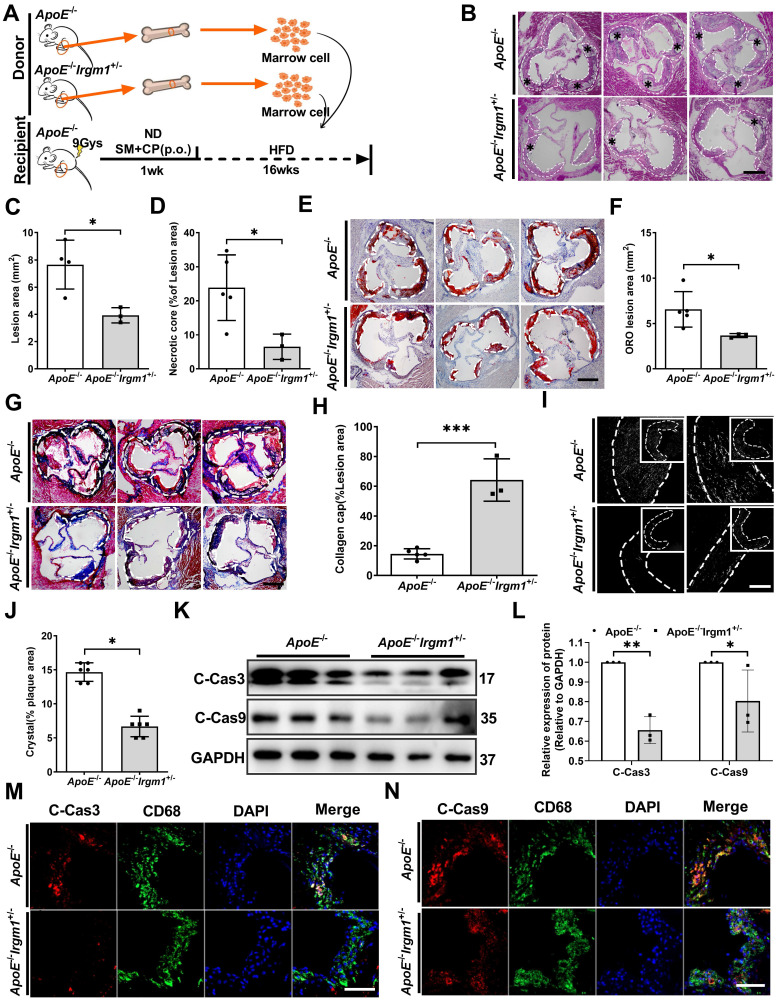
***Irgm1* deficiency in haematopoietic cells increases mouse atherosclerotic plaque stability and suppresses macrophage apoptosis. (A)** Schematic diagram for the establishment of the bone marrow chimera recipient mouse model. **(B)** Representative images for H&E staining of plaques in the aortic sinus from *ApoE*^-/-^ (n = 5) and *ApoE*^-/-^*Irgm1*^+/-^ (n = 3) recipients. A necrotic lipid core is indicated by *; dashed lines indicate the contour of the plaques; scale bars: 200 µm. **(C, D)** Lesion area and percentage of necrotic core areas in the plaques. **(E)** Representative images of neutral lipid in the aortic sinus detected by ORO staining from *ApoE*^-/-^ (n = 5) and *ApoE*^-/-^*Irgm1*^+/-^ (n = 3) recipients; scale bars: 200 µm.** (F)** Quantitative analyses of the ORO staining positive areas in the plaques. **(G)** Representative images of collagen in the aortic sinus detected by Masson staining from *ApoE*^-/-^ (n = 5) and *ApoE*^-/-^*Irgm1*^+/-^ (n = 3) recipients; scale bars: 200 µm. **(H)** Quantitative analyses of the percentage of collagen content in the plaques. **(I, J)** Representative images (I) and quantitative analyses (J) of cholesterol crystals from *ApoE*^-/-^ (n = 3) and *ApoE*^-/-^*Irgm1*^+/-^ (n = 3) recipients; scale bars: 100 µm. **(K, L)** Western blotting was used to detect protein levels of cleaved-caspase3/9 in the aortic arch from *ApoE*^-/-^ (n = 5) and *ApoE*^-/-^*Irgm1*^+/-^ (n = 3) recipients. GAPDH was used as a loading control. Quantitative data represent the fold change after normalised to GAPDH. (M, N) Representative images for co-location of cleaved-caspase3/9 (red) and CD68 (green) by immunofluorescence staining after 16 weeks of a high-fat diet in *ApoE*^-/-^ (n = 3) and *ApoE*^-/-^*Irgm1*^+/-^ recipients (n = 3); nuclei were stained with DAPI (blue). *ApoE*^-/-^ vs. *ApoE*^-/-^*Irgm1*^+/-^ recipients, *p < 0.05, **p < 0.01. Results are presented as the mean ± SD. Statistical analysis: unpaired Student's *t*-test.

To define the importance of *Irgm1* in haematopoietic cells in plaque stability, the bone marrow cells of *ApoE*^-/-^ or *ApoE*^-/-^*Irgm1*^+/-^ mice were transplanted into *ApoE*^-/-^ recipients. After one week of reconstitution, mice were switched to an atherogenic diet for the subsequent 16 weeks (Figure 7A). *Irgm1* mRNA decreased significantly in peritoneal macrophages isolated from recipients, indicating a complete change in the genotype of these cells to that of the donor cells (Figure S7A). As expected, compared with the control mice that received *ApoE*^-/-^bone marrow, the lesion area and ratio of NC in *ApoE*^-/-^*Irgm1*^+/-^ bone marrow chimera were significantly reduced (Figure 7B-D). Meanwhile, the absence of *Irgm1* in marrow-derived cells significantly decreased lipid content (Figure 7E, F), while increased collagen content (Figure 7G, H). Subsequently, when received bone marrow cells from *ApoE*^-/-^*Irgm1*^+/-^ mice, cholesterol crystals were significantly reduced, compared to control mice that received *ApoE*^-/-^ bone marrow (Figure 7I, J). Taken together, these data suggest that *Irgm1* deficiency in haematopoietic cells increases plaque stability.

Furthermore, we verified the effects of* Irgm1* deficiency in haematopoietic cells on macrophage apoptosis *in vivo*. Results showed that *Irgm1* deficiency in bone marrow cells significantly decreased the cleaved-caspase3 and cleaved-caspase9 ratio in atherosclerotic plaques (Figure S7B-D), which was consistent with the western blot results (Figure 7K, L). Finally, compared with the control mice that received *ApoE*^-/-^ bone marrow, the ratio of co-localization of cleaved-caspase3/9 with CD68 in the plaques of *ApoE*^-/-^*Irgm1*^+/-^ bone marrow chimera was significantly reduced (Figure 7M, N, Figure S7E, F). These results indicate that *Irgm1* deficiency in haematopoietic cells inhibits macrophage apoptosis in atherosclerotic lesions.

## Discussion

Here, we have demonstrated that IRGM and PR strongly correlate in STEMI patients. *In vivo* experiments further confirmed that *Irgm1* deficiency increases atherosclerotic plaque stability and macrophage apoptosis. In addition, *Irgm1* deficiency suppresses apoptosis in macrophages induced by MAPK signaling pathway activation, and ROS act as an upstream event. Our research reveals a novel view that *IRGM/Irgm1* regulates macrophage apoptosis and expands our knowledge of their role in regulating plaque stability.

In our previous studies, we found that *IRGM/Irgm1* promotes ox-LDL uptake by macrophages 20, as well as macrophage polarization to the pro-inflammatory M1 phenotype 21 during the initial phase of atherosclerosis. However, more importantly, rupture at the site of a vulnerable atherosclerotic plaque is the most frequent cause of acute coronary syndromes 45. However, surgical or percutaneous revascularization currently used in clinical does not address the basic biology of coronary atherosclerosis and therefore may have little effect on plaque stability 45. Therefore, identification and stabilization of vulnerable plaques are still important new directions in the treatment of coronary atherosclerosis. Macrophage survival is significantly correlated with plaque stability 9, 46. Therefore, it is necessary to investigate whether *IRGM/Irgm1* impact macrophage survival and plaque stability during advanced disease stages. In the current study, after feeding mice a high-fat to induce advanced atherosclerosis, *Irgm1*-deficient mice and bone marrow* Irgm1*-deficient chimeras showed typically stable plaques. Notably, in STEMI patients, TC and LDL-C in the RP group were significantly higher than those in the non-RP group. However, both before and after 16 weeks high-fat diet, there was no significant difference in serum lipid levels of *ApoE*^-/-^*Irgm1*^+/-^ mice compared with *ApoE*^-/-^ mice. Therefore, *IRGM/Irgm1* may not regulate plaque stability by altering blood lipid levels, however, analysis of the use of combined lipid-lowering drugs is still needed for further verification.

Recent research has focused on the emerging role of *IRGM/Irgm1* in coordinating autophagy 47. Oxidised low-density lipoprotein can induce autophagy to protect cells 8, 48-51, which is supported by our *in vitro* study results. After ox-LDL stimulation for 3 h, *IRGM* promotes autophagic flux complication in macrophage. Unexpectedly, after ox-LDL stimulation for 48 h, regardless of whether *IRGM/Irgm1* expression is modified, macrophages exhibited blocked autophagy flux. Studies have pointed out that the autophagy function in the advanced stage of AS is often impaired, which coincides with our *in vitro* results 52-54. Therefore, we believe that the effect of *IRGM/Irgm1* on macrophage autophagy does not explain the changes associated with atherosclerotic plaque stability in advanced stages. Moreover, increased macrophage apoptosis in advanced lesions 55, leads to enhanced inflammatory cascades, NC expansion, and increased PR risk 56, 57. In our study, in response to 48 h ox-LDL stimulation *in vitro, IRGM/Irgm1* deficiency significantly reduced the expression of apoptosis-related proteins. Furthermore, *ApoE^-/-^Irgm1*^+/-^ mice exhibited reduced macrophage apoptosis in plaques. Therefore, our results revealed a novel view that differs from regulating autophagy. *IRGM/Irgm1* can regulate macrophage apoptosis, which may explain the changes in plaque stability within advanced disease stages.

MAPK signaling controls macrophage apoptosis 58-60. Previous studies have shown that MAPK signaling can induce caspase8 activation and apoptosis via death-receptor pathway 61. However, it can also induce caspase9 activation and apoptosis via the mitochondria-dependent pathway which is also dependent on Bcl-2 family members 62, 63. Subsequently, both of these pathways induced the activation of caspase3, which leads to DNA fragmentation and morphological changes 64, 65. In our study* in vitro*, knockdown of *Irgm1* significantly decreased MAPK signaling activation. Subsequently, Bcl-2 increased, while cleaved-caspase3/9 decreased. Furthermore, si-*Irgm1* exhibited a synergistic anti-apoptotic effect with MAPK inhibitors. These results suggest that the mitochondrial pathway activated by MAPK is responsible for the regulation of apoptosis by *IRGM/Irgm1*. However, recent studies have reported the mitochondrial as the junction of the apoptotic pathway, with caspase9 also capable of inducing caspase8 activation 66. Therefore, we cannot overlook the possibility that IRGM/*IRGM1* regulates the death-receptor pathway and caspase8 activation, however, further verification is required. In addition, ERK activation is generally considered to be anti-apoptotic 67. However, si-*Irgm1* reduced p-ERK in our results, which seems contradictory. In fact, *Irgm1* may play a more dominant role in the regulation of JNK and p38. In addition, certain studies have indicated that ERK activation can also exhibit pro-apoptotic effects 68. These above conditions may explain our results. The second messenger ROS regulates MAPK activation via positive feedback, which is a common mediator in the pathogenicity of established cardiovascular risk factors and evokes many intracellular events including apoptosis 69, 70. In our *in vitro* studies, a positive feedback was observed. That is, *IRGM/Irgm1* regulates ROS production, MAPK activation and apoptosis. Meanwhile, *IRGM/Irgm1* deficiency reduces ROS production and acts as an upstream factor to inhibit MAPK activation and macrophage apoptosis.

The generalizability of these results is subject to certain restrictions. As a retrospective study, clinical data reflects the current epidemiology, serum, and plaque characteristics of STEMI patients in China. Therefore, it may not be suitable for patients with non-ST elevation ACS or patients from other ethnicities. It is, therefore, necessary to expand the study cohort or introduce a verification cohort for subsequent analysis. Second, considering that *Irgm1*^-/-^ mice with a C57BL/6 background may be embryonic lethal, in this study, we were only able to use *ApoE*^-/-^*Irgm1*^+/-^ mice to verify the effect of *Irgm1* deficiency *in vivo*. However, by transfecting si-*Irgm1 in vitro* and verifying the knockout efficiency, we provided further evidence to support the *in vivo* results. Third, it may be important to study the progression of atherosclerotic plaques by establishing an atherosclerosis model through a high-fat diet. However, this model may not perfectly characterize PR. Nevertheless, we are seeking to establish a PR model. Overall, despite these limitations, based on our methods' diversity and complementarity, we believe in the reproducibility of our results.

In conclusion, our study suggests that *IRGM* may be a potential PR predictor, which may provide a potential new target to treat and prevent PR-related acute cardiovascular events and, thus, has great clinical translation value.

## Methods

### Study participants

135 patients who were diagnosed with STEMI at the 2nd Affiliated Hospital of Harbin Medical University (Harbin, China) between August and December 2016 and who underwent OCT after thrombus aspiration were enrolled. Main exclusion criteria were angina pectoris, myocarditis, heart failure, aortic dissection, acute and chronic infections and infectious diseases, liver and kidney insufficiency, and severe progressive diseases. Ultimately, 85 patients with STEMI were enrolled. Patients were divided into two groups: patients with PR (PR group; n = 52) and without PR (non-PR group; n = 33). 43 additional healthy volunteers were also recruited. We analysed OCT images and obtained the serum of these 85 patients and 43 healthy volunteers. The definition of STEMI was in accordance with the 2015 ACC/AHA/SCAI guidelines published previously 71.

### OCT image acquisition and analysis

The frequency-domain OCT ILUMIEN system, along with the Dragon fly catheter (St. Jude Medical, Westford, Massachusetts, USA) were used to acquire OCT imaging of culprit lesions, as shown previously 72. Two experienced independent investigators (H.C and S.S) analysed OCT images according to the criteria of the Consensus OCT Document 73 using a proprietary OCT review workstation (St. Jude Medical, Westford, MA, USA) at an intravascular imaging and physiology core lab. If these two observers disagreed, a consensus reading was obtained by another investigator. Investigators were blinded to the subject information. PR was defined as a lipid plaque with fibrous cap continuity destroyed plus clear cavity formation within the plaque. The FCT covering the lipid core was measured three times at the thinnest location, and the mean value of triplicate measurements was calculated. TCFA was a lipid plaque in the presence of FCT < 65 μm with a maximum lipid arc of at least 90° 74.

### Patient blood vessel specimens

Atherosclerotic middle artery vessels were obtained from patients with arterial occlusion in the Department of Vascular Surgery of the First Affiliated Hospital of Harbin Medical University. Normal vessels were obtained from five patients who underwent lower limb amputation. Blood vessel specimens from patients were transported to the laboratory by dry ice and maintained at -40 °C after isolation. Blood vessel specimens were embedded in paraffin and sectioned consecutively for H&E and immunohistochemical staining.

### Animals

*Irgm1*^-/-^ mice (C57BL/6 background) were derived from a published source 21. *Irgm1*^-/-^ mice and *ApoE*^-/-^ mice were crossed to obtain *ApoE*^+/-^*Irgm1*^+/-^ mice. We screened *ApoE*^-/-^*Irgm1*^+/-^ mice by crossing *ApoE*^+/-^*Irgm1*^+/-^ mice. Due to the probability of embryonic lethality in *ApoE*^-/-^*Irgm1*^-/-^ mice, offspring of self-crossed *ApoE*^-/-^*Irgm1*^+/-^ mice were genetically identified. After PCR genotyping, we then established a model of advanced atherosclerosis in mice fed high-fat diet for 16 weeks. Anaesthesia was maintained with 1% isoflurane delivered in oxygen.

### Reagents

See Supplemental Tables 3-4.

### Bone marrow chimera recipient mice

*ApoE*^-/-^ (6-8-weeks-old) recipient mice were supplemented with fradiomycin, polymyxin, and sterile pH 3.0 water for a week, prior to irradiation with 9 Gy of radiation. The recipient mice were divided into two groups: received bone marrow macrophages (5 × 10^6^) from *ApoE*^-/-^ donor mice and *ApoE*^-/-^*Irgm1*^+/-^ donor mice by tail vein injection. Four months after bone marrow transplantation (BMT), peritoneal macrophages were collected for PCR analysis of bone marrow reconstitution.

### Cell culture

Human THP-1 cells and murine Raw264.7 cells were obtained from China Center for Type Culture Collection. The cell lines used in this study were authenticated using short tandem repeat (STR) analysis and regularly tested for mycoplasma. Raw 264.7 cells were cultured in Dulbecco's Modified Eagle's medium (DMEM) (SH30022.01B, HYCLONE, USA) containing 10% fetal bovine serum (0500, ScienCell, USA). THP-1 cells were cultured in 1640 medium (88365, Gibco, USA) supplemented with 10% fetal bovine serum (0500, ScienCell, USA). Cells were seeded in 60-mm dishes or 6- and 12-well plates and collected at 60%-80% confluency.

### Transfection

*IRGM* gene was constructed into pRP[Exp]-NeoR-CMV>MSC. Shuttle empty vector (Mijia Biotech, Beijing, China). Transfection was accomplished using Lipo3000 (L3000008, Invitrogen, USA) according to manufacturer's protocols with the following siRNA duplexes: negative control in RAW264.7 cells, 5'-CGUACGCGGAAUACUUCGAUU-3'; *Irgm1*-siRNA, 5'-GGGCUGGGAUUCUGUCAUA-3'; negative control in THP-1 cells, 5'-UUCUUCGAACGUGUCACGUTT-3'; *IRGM*-siRNA, 5'-GAGGTGATCTCTAACATCA -3'. Real-time qPCR was utilised to confirm efficiency of transfection.

### ROS detection

ROS generated by ox-LDL treatment in cells was evaluated by fluorescence intensity of the dihydroethidium (DHE) and 5-(and-6)-chloromethyl-2',7' -dichlorodihydrofluorescein diacetate (DCFH-DA) probe. Following the intake of ox-LDL, macrophages were harvested, washed, and incubated with 5 μM DHE probe (S0063, Beyotime, China) or 5 mg/mL DCFH-DA probe (S0033S, Beyotime, China) for 30 min at 37 °C in the dark. After rinsing, fluorescent signals were immediately measured using a fluorescence microscope and FACS Verse flow cytometer (BD, USA).

### Real-time quantitative PCR (RT-qPCR)

Total RNA extracted by TRIzol reagent (Thermo Fisher, USA) was reverse-transcribed utilising the RT Easy II First Strand cDNA Synthesis Kit (04379012001, Roche, Switzerland). We amplified cDNA (18 ng) in a Real-Time PCR Easy (SYBR Green I) (HY-K0501, MCE, China) on Bio-RAD Sequence Detection system (Bio-RAD, USA). The following primers were used in Table S5, gene expression values were normalised against β-actin.

### Pathological staining

Hearts were perfused with PBS and placed in a mould containing tissue-freezing medium and frozen. Sections were cut from the caudal of the aortic sinus to the proximal aorta. Slides were fixed with 4% paraformaldehyde and then stained with H&E, ORO, and Masson staining as previously described 21.

### Immunofluorescence

Frozen slides were rewarmed at room temperature (RT) for 10 min, and then fixed with cold acetone (-20 °C) for 10 min. After rinsing thrice in distilled water, slides were first incubated with 0.3% Triton-X 100 for 30 min at 37 °C and then with primary antibody at 4 °C overnight. On day 2, after rewarming for 10 min at RT, slides were incubated with secondary antibody at RT for 1 h. Finally, nuclei were stained with 0.5 g/L DAPI for 10 min and images were captured using a confocal laser scanning microscope (ZEISS LSM 700).

### Immunohistochemistry

Frozen slides were rewarmed at RT for 10 min, and then fixed with cold acetone (-20 °C) for another 10 min. After three rinses in distilled water, slides were incubated with 0.3% Triton-X 100 for 30 min at 37 °C. Endogenous peroxidase was blocked with 3% hydrogen peroxide, and slides were incubated with primary antibody at 4 °C overnight. The following day, frozen slides were rewarmed for 10 min at RT and then incubated with secondary antibody at RT for 1 h. Next, slides were exposed to 3,3'-diaminobenzidine (DAB) (ZSGQ-BIO, China) stain for 1-10 min, followed by Hematoxylin stain. Images were taken using ordinary forward microscopy (Olympus, BX41). Paraffin sections were first dewaxed and then fixed with 4% PFA for 5 min. After rinsing with PBS, the sections were incubated with 0.3% Triton-X 100 at 37 °C for 30 min, and then antigen repair proceeded.

### Western blotting

Total protein was extracted using RIPA buffer with phenylmethylsulfonyl fluoride, and Halt Protease and Phosphatase Inhibitor. Protein concentration was measured by a bicinchoninic acid protein assay. Protein samples (30 μg) were separated by sodium dodecyl sulfate-polyacrylamide gel electrophoresis (SDS-PAGE, P0015, Beyotime, China) and transferred to 0.22 μm PVDF membranes. After blocking with 5% dried skimmed milk for 2 h at RT in Tris-buffered saline, the membranes were probed with primary antibodies (1*:*1000) and incubated overnight at 4 °C. Membranes were incubated for 2 h in the presence of horseradish peroxidase (HRP)-conjugated secondary antibodies (1*:*8000) at RT. Immunoreactivity was visualised by chemiluminescence using a ChemiDoc^TM^ MP Imaging System (Tanon, China). Protein bands were quantified using a Bio-Rad Chemi EQ densitometer and Bio-Rad Quantity One software (Tanon, China) and normalised to GAPDH.

### Caspase3/9 and MMP2/9 Activity

Caspase3/9 activity in the vascular tissue or cell homogenates were evaluated using the Caspase3 Assay Kit (Solarbio, BC3830) or Caspase9 Assay Kit (Solarbio, BC3890) according to manufacturer's instructions. MMP2/9 activity in the vascular tissue were evaluated using the MMP2 Assay Kit (GMS50070.5, GENMED SCIENTIFICS INC, U.S.A) or MMP9 Assay Kit (GMS50074.5, GENMED SCIENTIFICS INC, U.S.A) according to manufacturer's instructions.

### Genotyping

Excised mouse toes were added to 100 μL tissue digestion solution (bimake) and digested for 15 min at 55 °C, then incubated for 5 min at 95 °C to inactivate enzymes. Tubes were centrifuged at 12,000 rpm for 5 min, with supernatant used as the PCR template (1000 ng). Primer sequences are listed in Table S6. Next, PCR amplification was performed, followed by agarose gel electrophoresis and imaging analysis using a gel imaging system (BIO-RAD, GelDoc Go).

### Autophagy with double-labelled adenovirus

IRGM expressions were modified by specific siRNA (knocked-down) or IRGM plasmid (knocked-in) in THP-1 cells for 24 h. Raw 264.7 cells were transfected with si-*Irgm1* for 24 h. Adenovirus vectors encoding LC3 (HBAD-mRFP-GFP-LC3, HANBIO, China) and ox-LDL (50 μg/mL) were added for 3 or 48 h. Transfection efficiency was detected by confocal laser scanning microscope (ZEISS LSM 700). Autophagosomes were represented by the co-localised yellow fluorescence of both GFP and RFP. Due to quenching of GFP signal in acidic compartments, stronger red fluorescence and less co-localisation in autolysosomes is observed. LC3 activation was represented by punctate dots in GFP-LC3 transfected cells.

### Detection of apoptotic cells by flow cytometry

Cells were cultured and incubated in a 5% CO_2_ atmosphere for 48 h at 37 °C and then harvested, washed twice with pre-chilled PBS, and resuspended in 200 µL of 1× binding buffer. The cells were stained with fluorescein isothiocyanate (FITC)-conjugated annexin V and propidium iodide (PI) using the Annexin V-FITC & PI Apoptosis Detection Kit (556570, BD Biosciences, USA).

### Statistical analysis

Statistical analyses were performed using SPSS version 20.0 (IBM, Armonk, NY USA) or GraphPad Prism 7.0. Continuous variables were expressed as mean ± SD for normally distributed variables and as median (25, 75^th^ percentiles) for non-normally distributed variables, and assessed by Student's *t*-test or Mann-Whitney U test, respectively. Categorical variables were presented as counts and percentages, and the comparisons were performed using a Chi-square test or Fisher's exact-test. A series of multiple logistic regression models were used to assess the relationship between the risk of PR and plasma IRGM levels. A 2-sided *p* value < 0.05 was considered significant. All data were analysed by an independent statistician.

### Study approval

This study complies with the basic principles of the* Declaration of Helsinki* and was approved by the Ethics Committee of the 2nd Affiliated Hospital of Harbin Medical University (Harbin, China). All patients provided written informed consent. Animals were euthanised by cervical dislocation. Animal experiments were performed according to guidelines approved by the Institutional Animal Care Committee and in compliance with the guidelines from Directive 2010/63/EU of the European Parliament with approval from the Harbin Medical University Ethics Review Board.

## Supplementary Material

Supplementary figures and tables.

## Abbreviations

ACS: acute coronary syndrome
DAMP: damage/danger-related molecular patterns
ERKs: extracellular signal-regulated kinases
HDL: high-density lipoprotein
IRGM: immunity-related GTPase family M protein
JNKs: c-Jun N-terminal kinases
NAC: N-acetylcysteine
NC: necrotic core
OCT: optical coherence tomography
ox-LDL: oxidised low-density lipoprotein
PR: plaque rupture
ROS: reactive oxygen species
STEMI: ST-segment elevation myocardial infarction
TC: total cholesterol
TCFA: thin fibrous cap atherosclerotic plaques
