# Supplementary material for: IRGM/Irgm1 facilitates macrophage apoptosis through ROS generation and MAPK signal transduction: Irgm1+/- mice display increases atherosclerotic plaque stability
Source: Theranostics. 2021 Sep 9;11(19):9358–75. doi: 10.7150/thno.62797 (PMC8490524; doi:10.7150/thno.62797)
Supplement: Supplementary file 1 — Supplementary figures and tables. [file thnov11p9358s1.pdf]

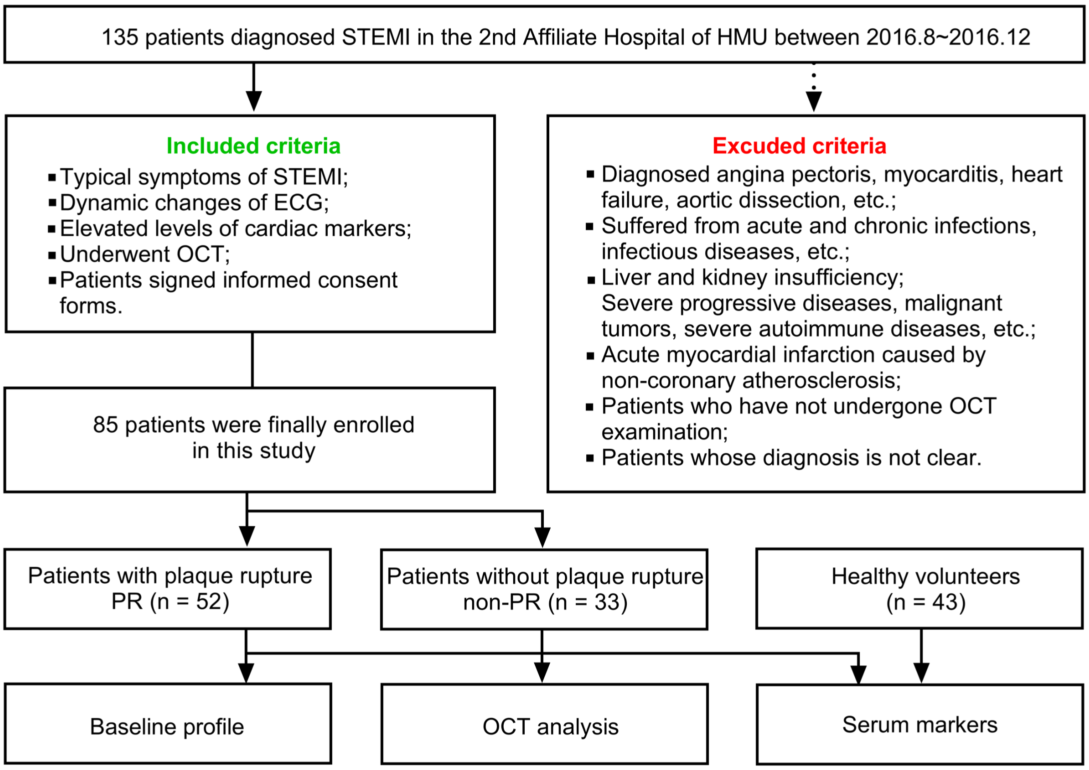

Supplemental Figure 1

STEMI, ST segment elevation myocardial infarction; HMU, Harbin Medical University; ECG, electrocardiogram; OCT, optical coherence tomography.

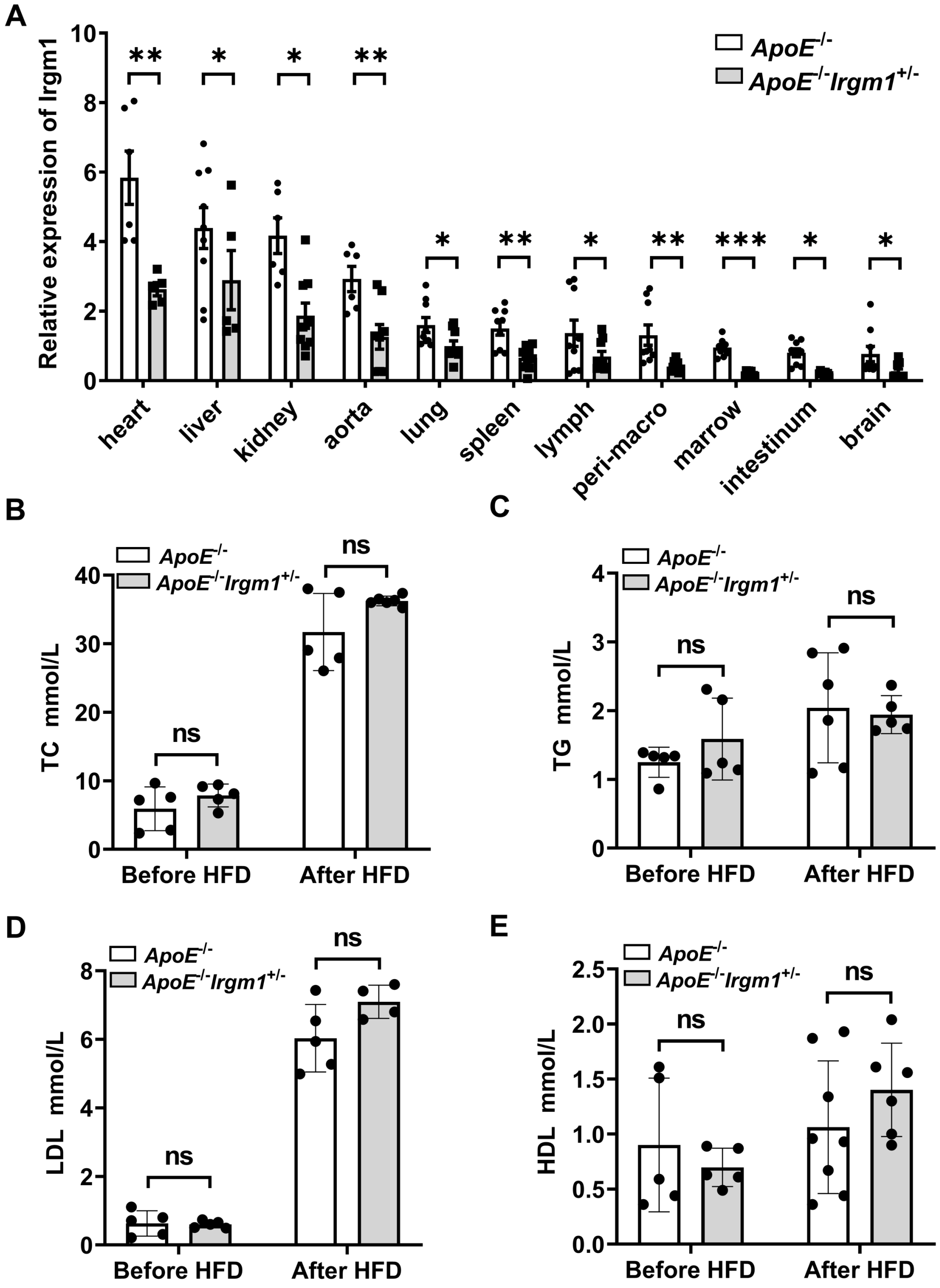

Supplemental Figure 2

(A) The expression of *Irgm1* in the heart, liver, spleen, lung, kidney, aorta, intestine, brain, lymph nodes, peritoneal macrophages, and bone marrow macrophages in *ApoE*$^{-/-}$ mice (n = 3) and *ApoE*$^{-/-}$*Irgm1*$^{+/-}$ mice (n = 3) as measured by qPCR. (B-E)

Quantitative analysis of the serum lipid levels in *ApoE*$^{-/-}$ and *ApoE*$^{-/-}$*Irgm1*$^{+/-}$ mice before and after a high-fat diet for 16 weeks. *$p < 0.05$, **$p < 0.01$, ***$p < 0.001$, ****$p < 0.0001$ ns = no significance. Results are presented as mean ± SD. Statistical analysis: unpaired Student's *t-test*.

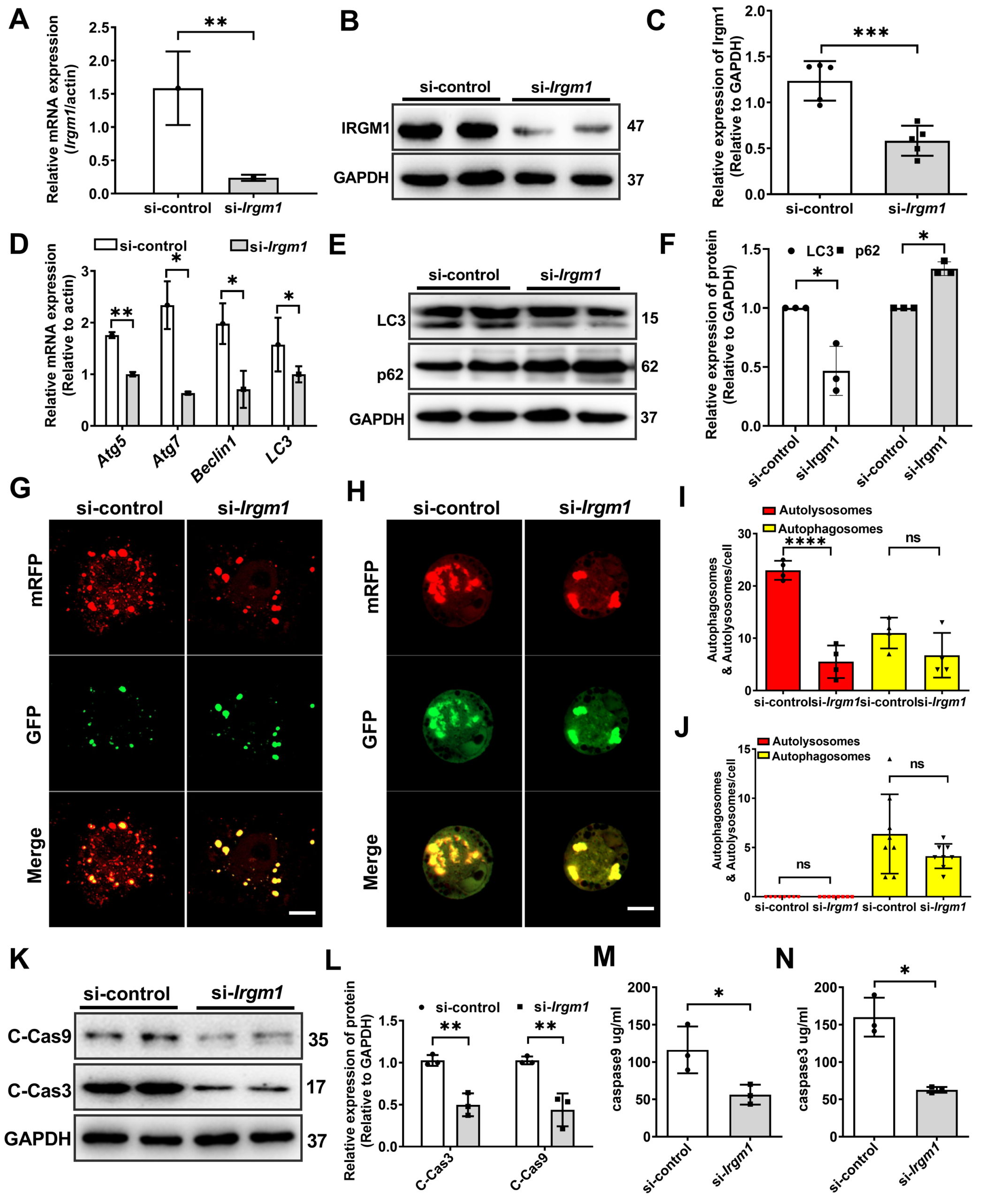

Supplemental Figure 3

(A-F) The Raw264.7 cells were stimulated with ox-LDL (50 μg/mL) for 3 h. The qPCR (A) and WB (B) were used to detect the silencing efficiency after transfection with si-*Irgm1*, and quantitative analysis results are shown (n = 3). (C) Duplicate samples were run in the same gels, with the membrane cut in half. Quantitative data

represent the fold change after normalized to GAPDH. (D) The expression of autophagy-related genes *LC3*, *Atg5*, *Atg7*, and *Beclin1* was detected by qPCR (n = 3). (E, F) The expression of autophagy-related proteins LC3 and P62 were detected by Western blot. (G) After the Raw264.7 cells were stimulated with ox-LDL (50 μg/mL) for 3 h, the autophagy double-labeled adenovirus fluorescent probe was used to detect the expression of autophagosomes (yellow) and autophagolysosomes (red). Green fluorescence was quenched in an acidic environment. Scale bar: 10 μm. (H) After stimulating Raw264.7 macrophages with ox-LDL (50 μg/mL) for 48 h, the autophagy double-labeled adenovirus fluorescent probe was used to detect the expression of autophagosomes (yellow) and autophagolysosomes (red). Green fluorescence is quenched in an acidic environment. Scale bar: 10 μm. (I) Quantitative analysis of the yellow and red puncta in (G). (J) Quantitative analysis of yellow and red puncta in (H). (K–N) Raw264.7 cells were transfected with si-*Irgm1* and then stimulated with ox-LDL (50 μg/mL) for 48 h. (K, L) Western blotting was used to detect the expression of cleaved-caspases3/9 (K), and quantitative analysis results are shown (L). Duplicate samples were run in the same gels, with the membrane cut in half. GAPDH was used as a loading control. Quantitative data represent the fold change after normalized to GAPDH. (M-N) Caspase3 (M) and caspase9 (N) activities were quantified.si-control vs. si-*Irgm1*, *$p < 0.05$, **$p < 0.01$, ****$p < 0.0001$. Results are presented as mean ± SD. Statistical analysis: unpaired Student's *t-test*.

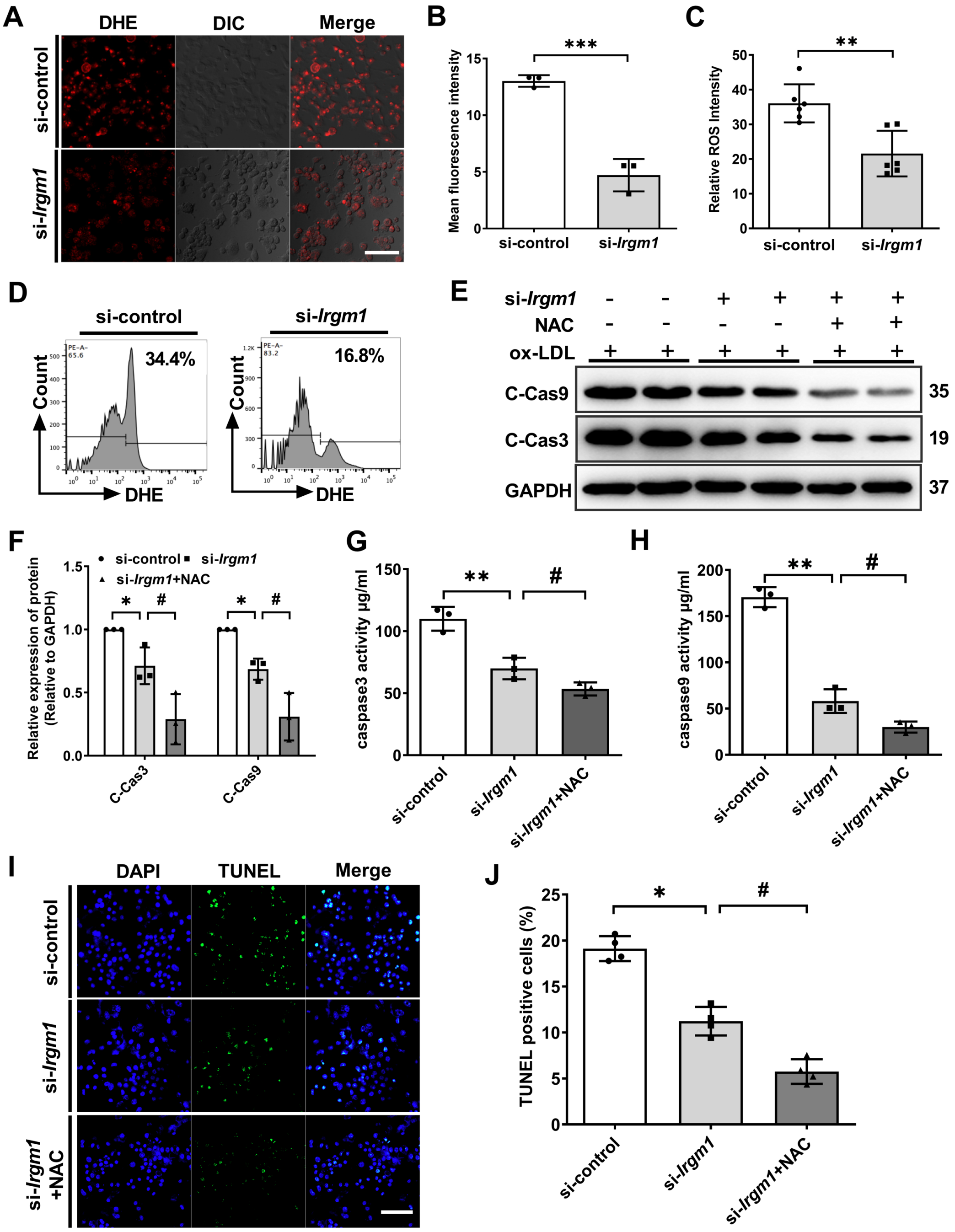

Supplemental Figure 4

(A-D) The Raw264.7 cells were transfected with si-*Irgm1* and si-control and then stimulated with ox-LDL (50 μg/mL) for 48 h. (A) Representative images of the reactive oxygen species labeled with DHE (5 μM) fluorescent probe were observed by confocal microscopy (n = 3 per group); scale bars: 50 μm. (B) Quantitative data in the

graph represent relative mean fluorescence intensity (MFI) (n = 3 per group). (C-D). Flow cytometry was used to detect ROS labeled with DHE fluorescent probes (n = 6 per group). (C) Quantitative data represent the percentage of DHE^+ macrophages. (E-J) Raw264.7 cells transfected with si-*Irgm1* and si-control, in the presence or absence of NAC, and then stimulated with ox-LDL (50 μg/mL) for 48 h. (E) Western blotting was used to detect the expression of cleaved-caspases-3/9. Duplicate samples were run in the same gels, with the membrane cut in half. GAPDH was used as a loading control. (F) Quantitative data represent the fold change after normalization to GAPDH. (G, H) Protein activities of caspases3/9 were detected by ultraviolet spectrophotometry (n = 3 in each group). (I-J) Representative images (I) and quantitative (J) for $TUNEL^+$ macrophages (green) by immunofluorescence. Nuclei were stained by DAPI (blue). DIC channel shown the contour of cells; scale bars: 50 μm; (n = 3 per group). si-control vs. si-*Irgm1*, *$p < 0.05$, **$p < 0.01$; si-*Irgm1* vs. si-*Irgm1* + NAC, #$p < 0.05$, ##$p < 0.01$. Results are presented as the mean ± SD. Statistical analysis: unpaired Student's *t-test*.

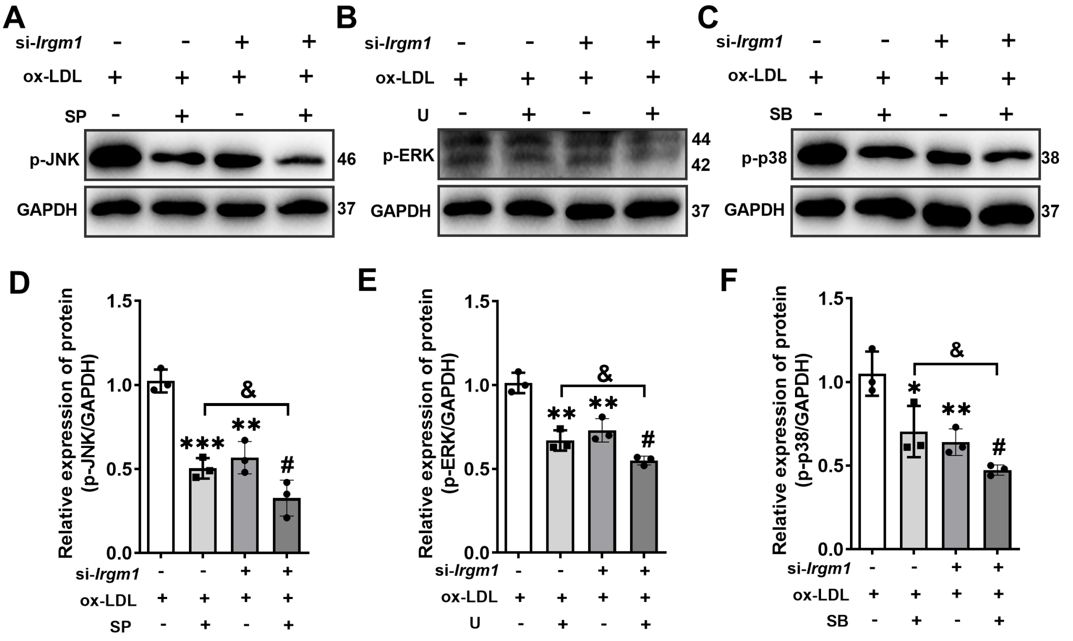

Supplemental Figure 5

(A-C) Representative images of the expression of p-JNK(A), p-ERK1/2(B) and p-p38(C) by western blot after the Raw264.7 cells were transfected with si-*Irgm1* and si-control, and then treated with SP600125 (10 μM), U0126 (10 μM) and SB203580 (10 μM) for 3 h combined with ox-LDL (50 μg/mL) for another 48 h. (D-F) Quantitative data of the expression of p-JNK (D), p-ERK1/2 (E) and p-p38 (F). GAPDH were used as loading control. Quantitative data represent the fold change after normalizing the band intensity of p-JNK, p-p38, p-ERK1/2, and *IRGM1* to GAPDH. vs. si-control + ox-LDL, **p* < 0.05, ***p* < 0.01, ****p* < 0.001, vs. si-*Irgm1* + ox-LDL, #*p* < 0.05, vs. inhibitor + ox-LDL, &*p* < 0.05. Results are presented as mean ± SD. Statistical analysis: unpaired Student's *t-test*.

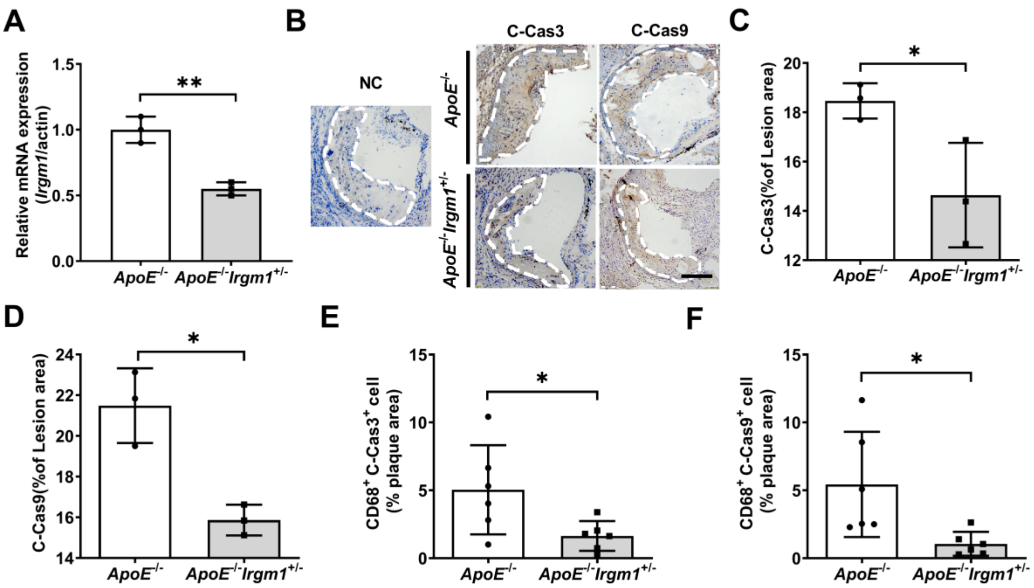

Supplemental Figure 6

(A) The expression of *Irgm1* in the peritoneal macrophages in *ApoE*$^{-/-}$ (n = 3) and *ApoE*$^{-/-}$*Irgm1*$^{+/-}$ recipients (n = 3) as measured by qPCR. (B) Representative images of cleaved-caspase3/9 in aortic sinus plaques. NC = negative control; scale bars = 100 µm. (C, D) Quantitative analyze of cleaved-caspase3/9 in aortic sinus plaques. (E, F) Quantitative data for co-location of cleaved-caspase3 (red) and CD68 (green) by immunofluorescence staining after 16 weeks of a high-fat diet in *ApoE*$^{-/-}$ (n = 3) and *ApoE*$^{-/-}$*Irgm1*$^{+/-}$ bone marrow chimera recipient mice (n = 3). *$p < 0.05$, **$p < 0.01$, ***$p < 0.001$, ns = no significance. Scale bar = 50 µm. Results are presented as mean ± SD. Statistical analysis: unpaired Student's *t-test*.

Supplemental Table 1. Clinical characteristics of patients for the present study

Covariate<br>No. of subjects	PR<br>52	non-PR<br>33	*P* value
Age, yrs	56.08±9.83	51.15±12.70	0.063
Male	39(75.0%)	27(81.8%)	0.462
DM	15(28.8%)	6(18.2%)	0.267
Smoking	30(57.7%)	25(75.8%)	0.089
Hypertension	30(57.7%)	13(39.4%)	0.301
Hyperlipidemia	22(42.3%)	15(45.5%)	0.776
LDL-C, mmol/L	3.29±0.90	2.82±0.94	0.039*
HDL-C, mmol/L	1.25±0.36	1.21±0.37	0.635
TG, mmol/L	1.79±0.79	1.56±0.84	0.258
TC, mmol/L	4.92±0.87	4.37±0.98	0.019*
hs-CRP, mg/dL	7.32±5.03	6.98±5.34	0.789
Peak-TnI, ng/mL	107.25±16.95	103.42±25.64	0.897

Values are expressed as Mean ± SD or n (%);

DM, diabetes mellitus; LDL-C, low-desity lipoprotein cholesterol; HDL-C, high-desity lipoprotein cholesterol; TG, triglycerides; TC, total cholesterol; hs-CRP, high sensitivity C-reactive protein; TnI, troponin I.

Supplemental Table 2. OCT findings of the patients for the present study.

Covariate	PR	non-PR	*P* value
No. of subjects	52	33	
TCFA	37(71.2%)	4(12.1%)	<0.001*
Min FCT, mm	0.05±0.02	0.07±0.03	0.026*
Lipid-rich plaque	46(88.5%)	12(36.4%)	<0.001*
Thrombus	48(92.3%)	31(93.9%)	0.775
Calcification	8(15.4%)	2(6.1%)	0.194
Cholesterol crystals	18(34.6%)	7(21.9%)	0.215

Values are expressed as Mean ± SD or n (%);

TCFA, thin-cap fibroatheroma; FCT, fibrous cap thickness.

Supplemental Table 3. Reagents or resources

REAGENT OR RESOURCE	SOURCE	IDENTIFIER
NAC	Beyotime	Cat#:S0077
ox-LDL	yiyuanBiotech	Cat#:YB-002
SB203580	Selleck	Cat#:S1076
U0126	Selleck	Cat#:S1102
Lipofectamine3000	Invitrogen	Cat#:L3000008
Mounting-medium	Dako	Cat#:CS703
SYBR Green qPCR Master Mix	MCE	Cat#:HY-K0501
SDS-PAGE	Beyotime	Cat#:P0015
PMSF	Beyotime	Cat#:ST505
Phosphatase inhibitor	Bimake	Cat#:B15001-A
Hypersensitive ECL chemiluminescence kit	HaiGene	Cat#:M2301
HE dye	Thermo	Cat#:67-63-0 (3%-5%)
Modified Oil Red O	Solarbio	Cat#:G1261
Modified masson's trichrome stain	Solarbio	Cat#:G1345
DHE fluorescence probe	Beyotime	Cat#:S0063
2',7'-Dichlorofluorescin Diacetate	Absin	Cat#:abs42197174
Skim milk powder	Biofroxx	Cat#:1172GR100
Albumin Bovine V	Biotopped	Cat#:MW68000
Triton X-100	Biofroxx	Cat#:1139ML100
DAPI	Beyotime	Cat#:C1002
TRIzol	HaiGene	Cat#:B0201
BCA	Beyotime	Cat#:P0012
Dual color prestained protein marker	EpiZyme	Cat#:WJ101
Caspase3 colorimetric assay kit	Solarbio	Cat#:BC3830
Caspase9 colorimetric assay kit	Solarbio	Cat#:BC3890

Supplemental Table 4. Antibodies

ANTIBODIES	SOURCE	IDENTIFIER
LC3	Sigma	Cat#:L7543
F4/80	Biolegend	Cat#:123113
CD11b	BD	Cat#:553312
p62	MBL	Cat#:PM045
cleaved-caspase3	CST	Cat#:ASP175
cleaved-caspase9	CST	Cat#:ASP353
MMP-2	Affinity	Cat#:AF5330
MMP-9	Affinity	Cat#:AF5228
GAPDH	ZSGB-BIO	Cat#:ta-08
p-ERK1/2	Wanleibio	Cat#:WL02368
CD68	Abcam	Cat#:ab53444
CD68	ZSGB-BIO	Cat#:ZM-0464
p-JNK	CST	Cat#:9251S
p-p38	CST	Cat#:4092S
bax	Abcam	Cat#:ab182733
bcl2	Abcam	Cat#:ab18858
IRGM	AbMart	Cat#:Clone:IG9
IRGM1	AbMart	Cat#:Clone:IC11
TRITC Goat Anti-Rabbit IgG H&L	Abcam	Cat#:ab6719
FITC goat anti-mice IgG	Abcam	Cat#:ab6785
FITC goat anti-rat	ZSGB-BIO	Cat#:ZF0315
FITC Annexin V	BD	Cat#:556547

Supplemental Table 5.

Gene	Forward primers (5'-3')	Reverse primers (5'-3')
Atg5	CTTCTGCACTGTCCATCTAAGG	ATCCAGAGTTGCTTGTGATCTT
Atg7	GCAGCCAGCAAGCGAAAG	CCGGTCTCTGGTTGAATCTCCTG
Beclin1	CCCGTGGAATGGAATGAGATTA	CCGTAAGGAACAAGTCGGTATC
LC3	AGAGTGGAAGATGTCCGGCT	CACTTCGGAGATGGGAGTGG

Supplemental Table 6.

**Gene**	**primers (5'-3')**
LRG1	GGAGAAAGTGAAGTACCC
LRG2	CTCTGACACCGAGAGAAT
Neo3	CATTTGTCACGTCCTGCA
